# Facile synthesis of three-dimensional structured carbon fiber-NiCo_2_O_4_-Ni(OH)_2_ high-performance electrode for pseudocapacitors

**DOI:** 10.1038/srep09277

**Published:** 2015-03-19

**Authors:** Wei Li, Lipeng Xin, Xin Xu, Qida Liu, Ming Zhang, Shujiang Ding, Mingshu Zhao, Xiaojie Lou

**Affiliations:** 1 Multi-disciplinary Materials Research Center, Frontier Institute of Science and Technology, Xi'an Jiaotong University, Xi'an 710049, China; 2 Department of Applied Chemistry, School of Science, Xi'an Jiaotong University, Xi'an 710049, China; 3 Department of Material Physics, School of Science, Xi'an Jiaotong University, Xi'an 710049, China; 4 A State Key Laboratory for Mechanical Behavior of Materials, Xi'an Jiaotong University, Xi'an 710049, China; 5 State Key Laboratory for Strength and Vibration of Mechanical Structures, Xi'an Jiaotong University, Xi'an 710049, China; 6 Shaanxi Province Key Laboratory of Advanced Functional Materials and Mesoscopic Physics, Xi'an Jiaotong University, Xi'an 710049, China; 7 MOE Key Laboratory for Nonequilibrium Synthesis and Modulation of Condensed Matter, Xi'an Jiaotong University, Xi'an. 710049, China

## Abstract

Two-dimensional textured carbon fiber is an excellent electrode material and/or supporting substrate for active materials in fuel cells, batteries, and pseudocapacitors owing to its large surface area, high porosity, ultra-lightness, good electric conductivity, and excellent chemical stability in various liquid electrolytes. And Nickel hydroxide is one of the most promising active materials that have been studied in practical pseudocapacitor applications. Here we report a high-capacitance, flexible and ultra-light composite electrode that combines the advantages of these two materials for pseudocapacitor applications. Electrochemical measurements demonstrate that the 3D hybrid nanostructured carbon fiber–NiCo_2_O_4_–Ni(OH)_2_ composite electrode shows high capacitance, excellent rate capability. To the best of our knowledge, the electrode developed in this work possesses the highest areal capacitance of 6.04 F cm^−2^ at the current density of 5 mA cm^−2^ among those employing carbon fiber as the conductor. It still remains 64.23% at 40 mA cm^−2^. As for the cycling stability, the initial specific capacitance decreases only from 4.56 F cm^−2^ to 3.35 F cm^−2^ after 1000 cycles under a current density of 30 mA cm^−2^.

The ever increasing demand for portable electronic equipment, and electric or hybrid electric vehicles, as well as the concerns of environmental pollution have stimulated the rapid development of the electro-chemical energy storage and conversion devices (EESCs). These devices typically include lithium-ion batteries and pseudo-capacitors^1^,^2^,^3^,^4^. In particular, pseudo-capacitors, as a typical representative of EESCs, have been widely studied due to their numerous merits, such as fast charge and discharge properties, long service life and high power density, wide range working temperature, high safety and the environmentally friendly feature^5^,^6^.

In the development of pseudo-capacitors, electrode materials are well known as one of the key factors affecting their electro-chemical performance^7^,^8^. So far, most of the investigations have focused on utilizing metals (e.g., Ni foil, Ni foam, Ti foil, Cu foil, etc.) as the current collector and transition metal oxides (e.g., NiO, NiOH, CoO, Co_4_O_3_, NiCo_2_O_4_, MnO_2_) as active materials to fabricate high-performance electrodes of pseudocapacitors^9^,^10^,^11^,^12^,^13^,^14^,^15^. These electrode materials were found to exhibit impressive pseudo-capacitive performance. Among all those materials, nickel hydroxide is a promising electrode material for pseudocapacitor applications due to its high theoretical capacitance (2082 F g^−1^ for Ni(OH)_2_ within 0.5 V) and good electrochemical reversibility in alkaline electrolyte, low costs, abundant natural resources, and excellent environment compatibility^16^,^17^,^18^,^19^. However, how to obtain Ni(OH)_2_ active material with excellent electrochemical performance and how to fabricate a high energy density and high power density electrode containing Ni(OH)_2_ remain big challenges^20^. In previous studies, Huang *et al.* found that Nickel−Cobalt Hydroxide-NiCo_2_O_4_ nanowires-Carbon fiber electrode was shown to possess a high areal capacitance of ~2.3 F cm^−2^ at a current density of 2 mA cm^−2^ 21. In addition, Xiao *et al.* reported that the NiCo_2_S_4_-Carbon fiber electrode exhibits a high discharge areal capacitance of 2.86 F cm^−2^ at 4 mA cm^−2^.^22^. Furthermore, Zhang *et al.* prepared two kinds of 3D hybrid nanostructure NiCo_2_O_4_ nanorods and ultrathin nanosheets electrode materials based on carbon nanofibers, the electrode shows excellent electrochemical performance due to its fine 3D hybrid nanostructure^23^. Recently, Nickel Cobalt Sulfide directly deposited on carbon fiber also shows high specific capacitance of 1418 F g^−1^ at 5 A g^−1^ with excellent rate capability^24^. Although these studies have very excellent achievements, it is still necessary and of great significance to unceasingly improve the electrode performances of pseudo-capacitors to meet the ever increasing social needs.

In this work, we designed and fabricated a unique 3D hybrid nanostructured electrode using the commercial two dimensional (2D) textured carbon fiber (CF) as the conductive substrate due to its good conductivity, ultra-lightness, excellent chemical stability and outstanding flexibility^25^,^26^,^27^. Then, needle-like NiCo_2_O_4_ was assembled uniformly and vertically on the surface of carbon fibers to form 3D hybrid structured CF-NiCo_2_O_4_ materials, the latter of which was found to have a better electrical conductivity compared to the binary metal oxides such as NiO and Co_3_O_4_ as reported in the previous studies^9^,^21^,^28^. Within the composite, the 3D hybrid structured CF-NiCo_2_O_4_ serves as a supporting material, while polycrystalline Ni(OH)_2_ was deposited on the surface of the NiCo_2_O_4_ needles by using an electrochemical deposition technology. In this way, a new ultra-light, flexible and porous 3D hybrid structured CF-NiCo_2_O_4_-Ni(OH)_2_ electrode was synthesized. The preparation process is shown in Fig. 1. Electrochemical measurements demonstrate that the 3D hybrid nanostructured CF–NiCo_2_O_4_–Ni(OH)_2_ electrode shows a high capacitance with excellent rate capability and good cycling stability.

## Results

The hydrothermally synthesized supporting material was firstly characterized by X-Ray Diffraction (XRD) (see Fig. 2a). All the diffraction peaks in the XRD pattern can be readily indexed as spinel NiCo_2_O_4_, according to the standard card (JCPDS Card No. 20-0781). The morphology of the as-prepared product was studied by scanning electron microscopy (SEM). The typical low-magnification SEM micrograph (Fig. 2b) clearly illustrates that the 3D hybrid nanostructured supporting material is composed of numerous needle-like NiCo_2_O_4_ with a sharp tip uniformly arranged on the CF surface. High-magnification SEM micrograph (Fig. 2c) reveals that the length of a NiCo_2_O_4_ needle is approximately 4 μm, and the diameter of the nano-needle ranges from being several nanometers at the tip of the needle to around 100 nm near the end of the needle. Bright-field transmission electron microscopy (TEM) images show that the samples are polycrystalline (Fig. 2d). High-resolution TEM micrographs (Fig. 2f) show the lattice fringes with interplane spacing of 2.34 nm, corresponding to the (222) plane of the spinel phase NiCo_2_O_4_ in agreement with our XRD results. Then, selected-area electron diffraction (SAED) measurements were carried out, and the results show that the NiCo_2_O_4_ needles have a polycrystalline structure, which is consistent with the observation in Fig. 2e. Therefore, we have illustrated that the well-defined 3D hybrid nanostructured CF-NiCo_2_O_4_ supporting materials can be obtained by using a facile, low-cost, effective solution route.

The polycrystalline Ni(OH)_2_ wrinkled nanosheets were subsequently deposited on the surface of the NiCo_2_O_4_ needles using a simple electrochemical deposition method. The total mass of the CF-NiCo_2_O_4_-Ni(OH)_2_ electrode is 17.29 mg, and that of the loaded Ni(OH)_2_ active material is 2.44 mg cm^−2^. The CF-NiCo_2_O_4_-Ni(OH)_2_ electrode was then analyzed using Raman spectroscopy. As seen in Fig. 3a, the Raman peaks at 185, 467, 508, and 651 cm^−1^, corresponding to F_2g_, E_g_, F_2g_, and A_1g_ modes of the NiCo_2_O_4_ nanowires, respectively^21^,^29^. Two new peaks are observed at 459 cm^−1^ and 534 cm^−1^ after electro-deposition, corresponding to the symmetric Ni–OH stretching mode and to the Ni–O stretching/vibrational mode, respectively^30^,^31^,^32^. In Fig. 3b and c, Ni(OH)_2_ was deposited uniformly on the surface and in the interspace of the 3D nanostructured supporting material. TEM micrographs show that the synthesized Ni(OH)_2_ is of a 3D wrinkled nanostructure consisting of densely wrinkled nanosheets (Fig. 3d, e). All the surface of the NiCo_2_O_4_ needles have been completely coated, indicating the formation of a highly porous 3D hybrid nanostructure. The corresponding SAED results (the inset of Fig. 3d) exhibit a diffraction ring pattern, indicating that the 3D wrinkled Ni(OH)_2_ is of a polycrystalline structure. The different diffraction rings can be readily indexed to the different crystal planes of the Ni(OH)_2_ phase. Fig. 3e illustrates that Ni(OH)_2_ is firmly connected to the surface of NiCo_2_O_4_ needles, which eventually forms a 3D wrinkled and porous nanostructure, consistent with the aforementioned SEM images analysis. The unique 3D hybrid porous nanostructure implies good electrochemical capacitive performance of the sample.

Then, the unique 3D hybrid porous structured CF-NiCo_2_O_4_-Ni(OH)_2_ product was used as the electrode material in three-electrode cell. Fig. 4a shows the typical cyclic voltammetry (CV) curves of the pseudocapacitor with the CF-NiCo_2_O_4_-Ni(OH)_2_ electrode at different scanning rates in the potential range from −0.2 V to 0.55 V. The CV curves clearly demonstrate that the CF-NiCo_2_O_4_-Ni(OH)_2_ electrode shows typical pseudocapacitive characteristics and excellent reversibility. In specific, a pair of representative redox peaks are clearly visible in each voltammogram at the scanning rate of 2, 4, 6, 8, and 10 mV, which is obviously distinct from the electric double-layer capacitance characterized by nearly rectangular CV curves. By comparing the CV curves with CF-NiCo_2_O_4_ supporting materials in Fig. 4b and by comparing the discharging curves of the CF-NiCo_2_O_4_-Ni(OH)_2_ electrode with CF-NiCo_2_O_4_ supporting materials at the current density of 5 mA cm^−2^ (Fig. S1), we can see that the CF-NiCo_2_O_4_-Ni(OH)_2_ electrode demonstrates better reversible and charging/discharging properties. The capacitive properties of the unique 3D hybrid porous structured CF-NiCo_2_O_4_-Ni(OH)_2_ electrode mainly originate from the strong faradaic redox reaction of polycrystalline Ni(OH)_2_ nanosheets. The capacitance of electrode was measured and could be correlated with the reversible reactions of Ni-O/Ni-O-OH by using the following equation^33^:
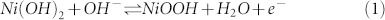


In Fig. 4a, the peak current increases significantly at the scanning rate from 2 mV to 10 mV, which indicates that the kinetics of the interfacial faradic redox reactions are rapid enough, which facilitates the transmission of electronic and ionic species. With increasing scan rates, the potential of the oxidation peak shifts in the positive direction and that of the reduction peak shifts in the negative direction, which is mainly related to the internal resistance of the electrode.

To further evaluate the application potential of 3D hybrid porous structured CF-NiCo_2_O_4_-Ni(OH)_2_ electrode, galvanostatic charge-discharge measurements were carried out between −0.1 and 0.45 V (vs. SCE) at various current densities, and the corresponding discharge curves of electrode are shown in Fig. 4c. The shapes of five curves are very similar and show ideal pseudocapacitive behaviour with sharp responses and small internal resistance drop. Moreover, there is a potential platform in every discharge curve. It is the typical pseudo-capacitance characterization of transition metal compounds that is in agreement with the result obtained from CV curves in Fig. 4a. This phenomenon is caused by a charge transfer reaction or electrochemical absorption–desorption process at the electrode–electrolyte interface. According to the formula (2), the calculated areal capacitance is shown in Fig. 4d. Encouragingly, the capacitances are 6.04, 5.72, 5.24, 4.56, 3.88 F cm^−2^ (corresponding to 2475, 2344, 2147, 1869, 1590 F g^−1^, respectively) when the dischare current densities are 5, 10, 20, 30 and 40 mA cm^−2^, respectively. To the best of our knowledge, the areal capacitances obtained from the CF-NiCo_2_O_4_-Ni(OH)_2_ electrode in this work are the highest values among those employing 2D textured carbon fiber as the conductor. The capacitance gradually decreases as the current density increases. The reason is that at a higher current density the incremental voltage drops and the active material involved in the redox reaction is insufficient. In comparison with the specific capacitance of 6.04 F cm^−2^ measured at the current density of 5 mA cm^−2^, the specific capacitance decreases to be 3.88 F cm^−2^ when the current density increases to 40 mA cm^−2^, but still remains 64.2% of the original value. These results illustrate that the CF-NiCo_2_O_4_-Ni(OH)_2_ electrode possessed excellent rate capability.

In addition, to investigate the stability of CF-NiCo_2_O_4_-Ni(OH)_2_ electrode, the cycling endurance of the electrode was evaluated by repeatedly charging-discharging measurements at a constant current density of 30 mA cm^−2^ and the results are shown in Fig. 4e. The areal capacitance is around 4.56 F cm^−2^ in the first cycle and it gradually decreases to 3.35 F cm^−2^ after 1000 cycles, corresponding to a 26.6% decrease in the initial specific capacitance. Although the cycling stability is not good enough, the capacitance of the CF-NiCo_2_O_4_-Ni(OH)_2_ electrode after 1000 cycles is still higher than the areal capacitance of the initial charging/discharging cycle reported in some recent studies, even at a heavy current density up to 30 mA cm^−2^ 21,22,24.

## Discussion

**Figure 1:**
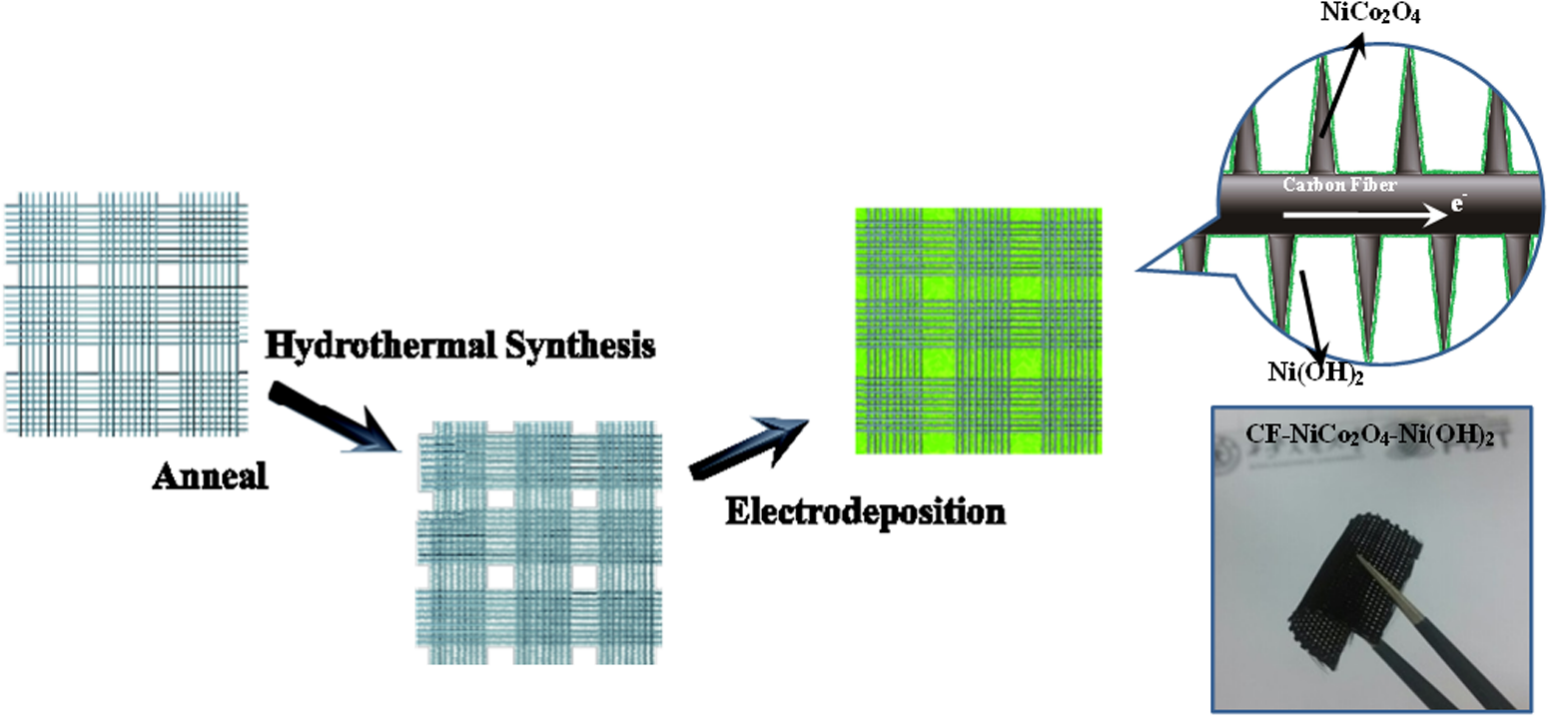
Schematic diagram of the preparation procedure of the CF–NiCo_2_O_4_–Ni (OH)_2_ 3D hybrid structured electrode material.

**Figure 2:**
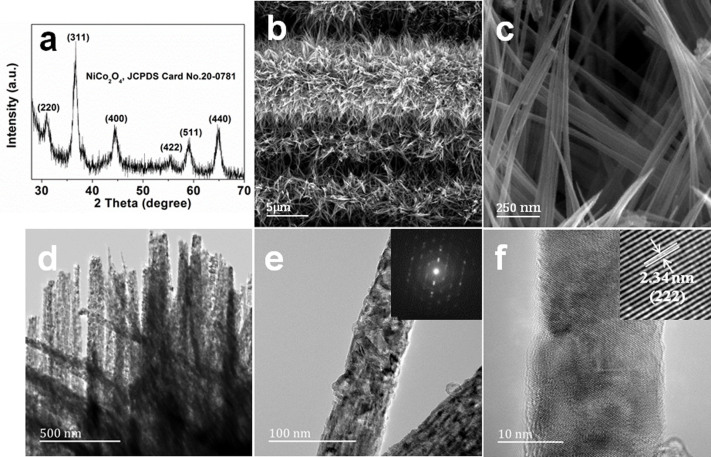
The structure and morphology characterization of the CF-NiCo_2_O_4_ supporting materials. (a) X-ray diffraction pattern of the CF-NiCo_2_O_4_ supporting materials; (b) Typical FESEM images of the CF-NiCo_2_O_4_ supporting materials after being annealed at 350°C for 2 h; (c) TEM bright field image of the assembled polycrystalline NiCo_2_O_4_ needles; (d) TEM image and SAED pattern of the NiCo_2_O_4_ needle; (e) HRTEM lattice pattern of the NiCo_2_O_4_ needles; (f) HRTEM micrograph of the NiCo_2_O_4_ needle.

**Figure 3:**
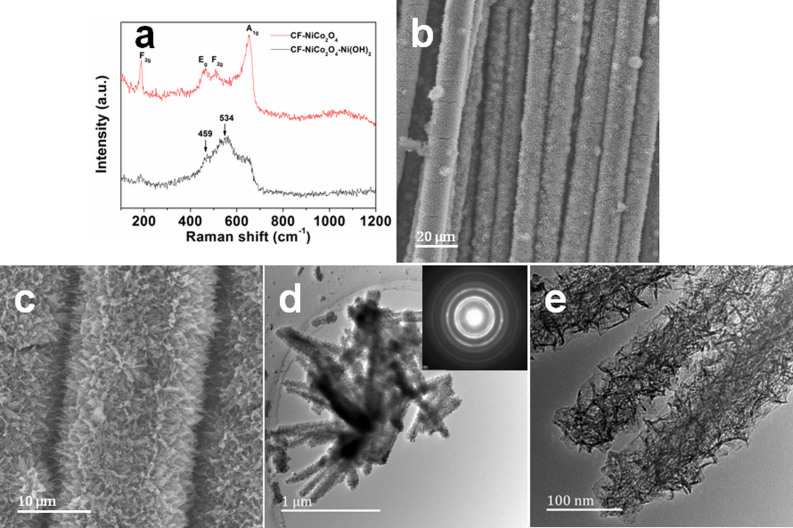
The crystal structure and morphology characterization of the CF-NiCo_2_O_4_-Ni(OH)_2_ electrode materials. (a) Raman spectra of CF-NiCo_2_O_4_, and CF- NiCo_2_O_4_-Ni(OH)_2_; (b, c) Typical SEM images of CF-NiCo_2_O_4_-Ni(OH)_2_; (c, d) TEM images of the 3D wrinkled polycrystalline Ni(OH)_2_ coated on the NiCo_2_O_4_ needles and the SAED pattern of Ni(OH)_2_.

The aforementioned electrochemical measurements demonstrate that the 3D hybrid nanostructured CF-NiCo_2_O_4_-Ni(OH)_2_ electrode material shows excellent pseudocapacitive behavior, such as higher capacitance, better rate capability and cycling stability. Note that the areal capacitance of this hybrid electrode is as high as 6.04 F cm^−2^ at the current density of 5 mA cm^−2^. The excellent electrochemical performance of the CF-NiCo_2_O_4_-Ni(OH)_2_ electrode material may be attributed to its 3D hybrid structure of multiscale, ranging from nanoscale to microscale. First, the 3D hybrid structure of the well-defined CF-NiCo_2_O_4_ supporting materials provide an efficient 3D electric-conductive network, which facilitates the high-speed electron transport and serves as the foundation for electro-deposited Ni(OH)_2_. Second, the 3D CF-NiCo_2_O_4_ supporting material also has more active surface area for accommodating more active material Ni(OH)_2_. Third, the 3D wrinkled framework of Ni(OH)_2_ nanosheets allows the facile penetration of electrolyte into the electrode, and promotes the surface redox reactions, which offers a relatively high electro-active surface area, as described in the schematic diagram in Fig. 1.

**Figure 4:**
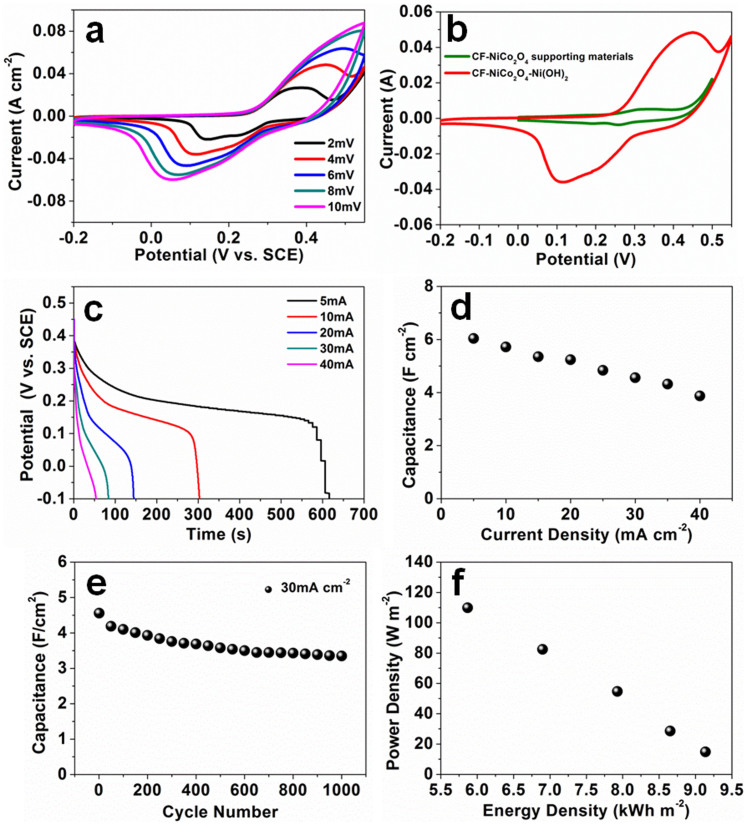
Electrochemical characterizations of the 3D hybrid nanostructured CF-NiCo_2_O_4_-Ni(OH)_2_ electrode. (a) CV curves at various scanning rates from 2 to 10 mV S^−1^; (b) CV curves comparison of the CF-NiCo_2_O_4_ supporting materials and CF-NiCo_2_O_4_-Ni(OH)_2_ electrode at scaning rate of 4 mV s^−1^ (c) Discharge voltage profiles at various current densities from 5 to 40 mA cm^−2^; (d) The change in capacitance with increasing current density; (e) The capacitance cycling performance at a constant current density of 30 mA cm^−2^; (f) Areal energy density and areal power density of CF-NiCo_2_O_4_-Ni(OH)_2_ electrode evaluated at different current density.

Energy density and power density are two key factors for evaluating the power applications of pseudocapacitors. A good pseudocapacitors can provide high energy density and capacitance. The power density and energy density were estimated from discharge curves by the formulas (3) and (4). As shown in Fig. 4f, the energy density of the CF-NiCo_2_O_4_-Ni(OH)_2_ electrode was able to reach 9.14 kWh m^−2^ at a power density of 14.83 W m^−2^, and still remained at 5.86 kWh m^−2^ at a power density of 109.9 W m^−2^. Compared with other 3D hybrid porous structures that use 2D textured carbon fiber as the current collector (e.g.: Nickel Cobalt Sulfide, Nickel–Cobalt Hydroxide Nanosheets,NiCo_2_S_4_ Nanotube) and are applied as electrode of pseudocapacitors, the CF-NiCo_2_O_4_-Ni(OH)_2_ electrode exhibited higher areal capacitance and energy density (See Table 1).

## Conclusions

To sum up, the 3D hybrid nanostructured CF-NiCo_2_O_4_ supporting materials were successfully synthesized by using a facile solution method and a simple annealing treatment. By using the 3D hybrid nanostructured CF-NiCo_2_O_4_ materials as the supporting materials, we have fabricated a new electrode material for pseudocapacitors via electrochemical deposition – the 3D hybrid nanostructured CF-NiCo_2_O_4_-Ni(OH)_2_. The total mass of the composite electrode was found to be 17.29 mg only. Electrochemical measurements demonstrate that the pseudocapacitor with the synthesized CF-NiCo_2_O_4_-Ni(OH)_2_ electrode exhibits high specific capacitance and excellent cycling stability. To the best of our knowledge, the CF-NiCo_2_O_4_-Ni(OH)_2_ electrode in this work shows the highest areal capacitance of up to 6.04 F cm^−2^ at the current density of 5 mA·cm^−2^, among those employing carbon fiber as the supporting substrate. The capacitance of the electrode still remains to be 3.35 F cm^−2^ at the current density of 30 mA·cm^−2^ after 1000 cycles. The excellent performance of the electrode material is attributed to the unique 3D hybrid micro/nano-structure of the CF-NiCo_2_O_4_-Ni(OH)_2_ electrode. The performance we achieved suggests that the 3D hybrid nanostructured electrode prepared using simple synthesis process and developed in this work have great potential in various energy storage technologies. Therefore, we expect that pseudocapacitors with better electrochemcial performance could be fabricated by using the 3D hybrid nanostructured electrode.

## Methods

All the reagents used in the experiments are of analytical grade and are used without further purification.

### The synthesis of the CF-NiCo_2_O_4_ supporting materials

In a typical procedure, 1 mmol Ni(NO_3_)_2_·6H_2_O, 2 mmol Co(NO_3_)_2_·6H_2_O, and 5 mmol urea were dissolved in 35 ml. deionized (DI) water. After stirring for about 10 min, a transparent pink-colored solution was obtained. The solution was then sealed in a Teflon-lined stainless autoclave. A piece of clean carbon cloth substrate of 3 × 7 cm^2^ in size was immersed into the reaction solution. Afterwards, the autoclave was put in an oven, and heated to 120°C and kept at that temperature for 5 h. After cooling down naturally to room temperature, the sample was collected and then washed with DI water and absolute ethyl alcohol for several times before it was dried in a vacuum oven at 80°C for 2 h. Finally, the dried sample was calcined at 350°C in the air for 2 h with the heating rate at 4°C min^−1^.

### The fabrication of the 3D hybrid nanostructured CF- NiCo_2_O_4_-Ni(OH)_2_

The CF-NiCo_2_O_4_ supporting materials were accurately weighed and cut into 1 × 1 cm^2^ pieces, before being employed as the work electrode in the 100 ml pure and transparent 0.1 M Ni(NO_3_)_2_ solution for a standard three-electrode cell at room temperature (about 25°C), where the Pt foil serves as the counter electrode and a saturated calomel electrode (SCE) as the reference electrode. Electro-deposition was performed at the potential of −0.9 V for 400 s. Then, the obtained sample was washed with DI water and ethanol for several times. The product was then dried at 100°C for 10 h in a drying oven. The mass of the CF- NiCo_2_O_4_-Ni(OH)_2_ and the pure Ni(OH)_2_ was 17.29 mg cm^−2^ and 2.44 mg cm^−2^, respectively, being weighed with a high precision analytical balance of Sartorius BT25S.

### Electrode materials characterizations and electrochemical measurements

X-ray diffraction (XRD) patterns were collected with a SHIMADZU XRD-7000 X-ray diffractometer in a highly intensive Cu Kα irradiation (Voltage: 40.0 Kv, Current: 30.0 mA, λ: 1.5406 Å). The morphology of the products was examined by field-emission scanning electron microscopy (Hitachi, SU-8010). TEM and HRTEM images were recorded on a JEOL JEM-2100F microscope that operates at 200 kV. Transmission electron microscope (TEM) images were taken through a JEOL-2100F microscope. The electrodeposited CF- NiCo_2_O_4_-Ni(OH)_2_ product was used as the working electrode with 2.44 mg Ni(OH)_2_ active material. The electrochemical tests were conducted in a CHI660D electrochemical workstation in an aqueous KOH electrolyte (2.0 M) with a three-electrode cell where Pt foil serves as the counter electrode and a saturated calomel electrode (SCE) as the reference electrode. The areal capacitance of the electrode materials can be calculated according to the following equation:
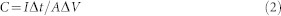
In which *I* is the discharge current (*A*), Δ*t* is the discharge time (s), Δ*V* is the voltage window (*V*) and *A* is the area of the electrode material (cm^2^).

The energy density (E) and power density (P) are important parameters for pseudocapacitor device. In this study, we also evaluated the energy density and power density of the Ni_3_S_2_/Ni electrodes from the discharge cycles as follows:
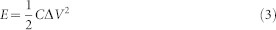

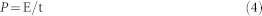


Where *C* is the areal capacitance (F m^−2^), t is the discharge time (s), and Δ*V* is the voltage window (*V*).

## Author Contributions

W. Li and X.J. Lou designed the project. W. Li performed the experiments, calculations and data analysis. L.P. Xin and X. Xu assisted with some of the experiments. S.J. Ding, M.S. Zhao and X.J. Lou guided the work and analysis. W. Li, Q.D. Liu, M. Zhang and X.J. Lou co-wrote the paper.

## Supplementary Material

Supplementary Information Supplementary Information

## Figures and Tables

**Table 1:** Areal capacitance comparison of different electrode materials that employ commercial 2D textured carbon fiber as the current collector

Active materials*	Current density	Areal capacitance	Energy density **
Nickel–Cobalt Hydroxide Nanosheets^21^	2 mA cm^−2^	2.3 F cm^−2^	3.48 kWh m^−2^
NiCo_2_S_4_ Nanotube^22^	4 mA cm^−2^	2.86 F cm^−2^	3.58 kWh m^−2^
Nickel Cobalt Sulfide^24^	4 mA cm^−2^	1.14 F cm^−2^	1.43 kWh m^−2^
Ni(OH)_2_ (in this work)	5 mA cm^−2^	6.04 F cm^−2^	9.14 kWh m^−2^

*The current collectors are all 2D textured carbon fiber.

**The energy density is calculated according to the data in the correspondent references and Formula (3).
