# Supplementary material for: Facile synthesis of three-dimensional structured carbon fiber-NiCo2O4-Ni(OH)2 high-performance electrode for pseudocapacitors
Source: Sci Rep. 2015 Mar 19;5:9277. doi: 10.1038/srep09277 (PMC4365399; doi:10.1038/srep09277)
Supplement: Supplementary Information [file srep09277-s1.doc]

Supplementary Information

**Facile synthesis of three-dimensional structured carbon fiber-NiCo_2_O_4_-Ni(OH)_2_ high-performances electrode for pseudocapacitors**

**Wei Li,^1,4*^ Lipeng Xin,^1,4^ Xin Xu,^2,4,7^ Qida Liu,^5^ Ming Zhang,^1,4^ Shujiang Ding,** **^2,4,7^ Mingshu Zhao,^3,4,6,7^ and Xiaojie Lou^1,4*^**

*^1^Multi-disciplinary Materials Research Center, Frontier Institute of Science and Technology, Xi’an Jiaotong University, Xi’an 710049, China*

*^2^Department of Applied Chemistry, School of Science, Xi'an Jiaotong University, Xi'an 710049, China.*

*^3^Department of Material Physics, School of Science, Xi'an Jiaotong University, Xi'an 710049, China.*

*^4^A State Key Laboratory for Mechanical Behavior of Materials, Xi'an Jiaotong University, Xi'an 710049, China.*

*^5^State Key Laboratory for Strength and Vibration of Mechanical Structures, Xi’an Jiaotong University, Xi'an 710049, China.*

*^6^Shaanxi Province Key Laboratory of Advanced Functional Materials and Mesoscopic Physics, Xi'an Jiaotong University, Xi'an 710049, China.*

*^7^MOE Key Laboratory for Nonequilibrium Synthesis and Modulation of Condensed Matter, Xi’an Jiaotong University, Xi’an, 710049, China.*

AUTHOR EMAIL ADDRESS: xlou03@mail.xjtu.edu.cn & liwei03453@stu.xjtu.edu.cn


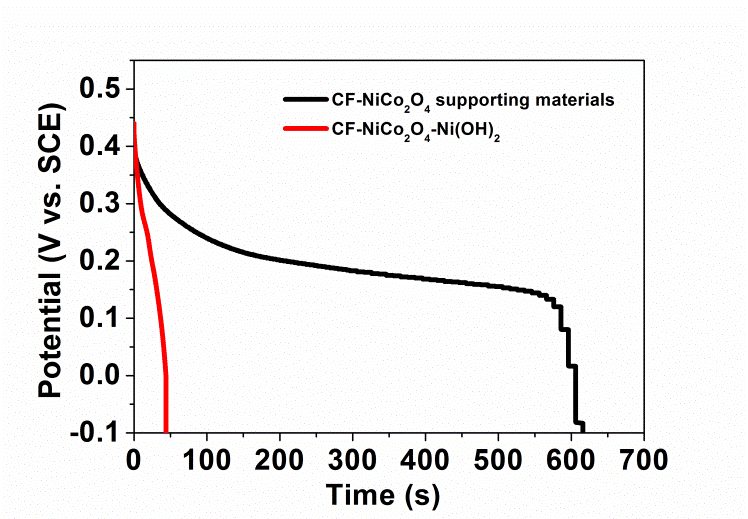


Fig. S1. The discharging curves of the CF-NiCo_2_O_4_ supporting materials and the CF-NiCo_2_O_4_-Ni(OH)_2_ electrode at the current density of 5 mA cm^-2^
